# Assessment the Changing Trend of Susceptibility to Two Insecticides among Field-Population *Culex quinquefasciatus* Compared with the Same Population Undergoing to Multiple Colonization

**DOI:** 10.18502/jad.v14i2.3736

**Published:** 2020-06-30

**Authors:** Atieh Shemshadian, Mohammad Reza Abai, Hassan Vatandoost, Navid Dinparast-Djadid, Mohammad Ali Oshaghi, Abdolrasoul Mojahedi

**Affiliations:** 1 Department of Medical Entomology and Vector Control, School of Public Health, Tehran University of Medical Sciences, Tehran, Iran; 2 Department of Chemical Pollutants and Pesticides, Institute for Environmental Research, Tehran University of Medical Sciences, Tehran, Iran; 3 Malaria and Vector Research Group, Biotechnology Research Center (BRC), Pasteur Institute of Iran, Tehran, Iran; 4 Provincial Health Center, Bandar Abbas University of Medical Sciences, Bandar Abbas, Iran

**Keywords:** *Culex quinquefasciatus*, Variation, Susceptibility, Insecticides multiple generations

## Abstract

**Background:**

During the past decade, rapid development of insecticide resistance have been reported among many species of mosquito vectors against four main categories of insecticides worldwide. The aim of the research was to assess the variation trend of susceptibility levels of *Culex quinquefasciatus* to two insecticides separately for the field population compared with subsequent generations of the same sample after multiple colonization.

**Methods:**

Larvae and pupae of *Cx. quinquefasciatus* were collected from house sewages and reared to adult which blood-fed on roosters. Ten percent sucrose fed female mosquitoes aged 2–3 days were used for susceptibility tests with DDT and deltamethrin. Susceptibility levels was assessed in the adult stage of field stran *Cx. quinquefasciatus* against DDT 4.0% and deltamethrin 0.05% and continued up to next six generations undergoing multiple rearing at insectary condition.

**Results:**

The susceptibility levels to DDT 4.0% did not change compared to the field with the lab population to six generations. Regarding deltamethrin 0.05%, no significant difference was shown between field strain (58.3%) and 3^rd^ generation (52.7%) compared to the 6^th^ one (33.8%).

**Conclusion:**

This finding may reflect the role of the kdr gene in resistance to organochlorine which has cross-resistance with pyrethroid insecticides. The results of this study clearly showed the irreversible trend of pyrethroid resistance among colonized mosquitoes. This is the first study of the resistance status of *Cx. quinquefasciatus* in Iran.

## Introduction

*Culex quinquefasciatus* Say, commonly known as the southern house mosquito, is widely distributed in the tropical and subtropical zones (1). It is also found at high densities in similar climates southern Iran. The biologic behavior of this species brings mosquitoes to the adjacent premises, providing conditions for easy transmission of arboviral diseases to humans as well as to domestic and wild animals, including West Nile fever, Saint Louis encephalitis, Western equine encephalitis, Rift Valley fever, avian malaria and lymphatic filariasis.

This mosquito also plays an important role in the transmission of the nocturnal periodic form of *Wuchereria bancrofti* (2) and is a major cause of acute and chronic morbidity, affecting all ages and both sexes throughout the tropical and subtropical areas of the world (3). Moreover, *Cx. quinquefasciatus* is identified as a potential vector of Zika virus (4). The major strategy for controlling this mosquito is application of insecticides. Additionally, this species can become quickly resistant to most insecticides compared to other mosquitoes (5) and resistant to different ones in most countries in the world (6). Resistance to DDT in *Cx. quinquefasciatus* was reported in West Africa for the first time (7). There are several reports of resistance to different groups of insecticides from West African regions including Côte d’Ivoire, Burkina Faso, Benin and Ghana (8–10). According to these reports, resistance to the organochlorine insecticides has developed by releasing pesticide residues into wastewater in major cities (11). Resistance of *Cx. quinquefasciatus* to pyrethroid was first reported from California (12). Resistance to organophosphates was reported in a strain of this vector in Sri Lanka due to increased esterase activity (13). There are also reports on resistance to a variety of insecticides from Thailand, Pakistan, France, and Saudi Arabia (14–16). However, there is no report of the resistance status of *Cx. quinquefasciatus* to insecticides in southern Iran.

The aim of the research was to assess variation trend of susceptibility levels of *Culex quinquefasciatus* to two insecticides separately for field population compared with subsequent generations of the same sample achieved after multiple colonization.

## Materials and Methods

### Mosquito collection

**Fig. 1.**
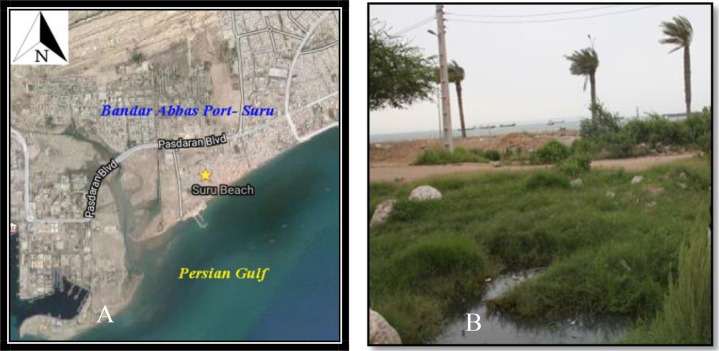
A. Satellite map of Suru location, Bandar-Abbas Port, Hormozgan Province, Persian Gulf littoral, Iran b. Ground landscapes of the breeding places of *Culex quinequefaciatus* in study area

The larval and pupae sampling of *Cx. quinquefasciatus* were done in Suru County, Bandar-Abbas District, Hormozgan Province in 27.168142° N and 56.252175° E at an elevation of 3m above sea level from March to April 2016 (Fig. 1). In order to provide laboratory colonies of mosquitoes, the larvae and pupae of *Cx. quinquefasciatus* were collected from breeding places in the wastewater of the houses running outside near the seaside using the dipping method (Fig. 1). Moreover, immature mosquitoes were collected from abandoned boats where the polluted water could penetrate. The eggs, larvae and pupae stages of *Cx. quinquefasciatus* along with their breeding places, water, and herbs were transported to an insectary located in Bandar-Abbas Training and Research Station. The mosquitoes were reared at 30±5 °C with 65–80% relative humidity. Female mosquitoes aged 2–3 days were fed on 10% sucrose solution and used for susceptibility tests.

### Insecticides

Susceptibility tests was done using with both DDT 4.0% impregnated-papers with impregnation date July 2013, expiry date July 2018 with Batch No under code: DD 188 and deltamethrin impregnated-papers with impregnation date July 2016, expiry date July 2017 with Batch No under code: DE 332. The impregnated-papers to insecticides was purchased from the Vector Control Research Unit, School of Biological Sciences, 11800 Minden, Penang, Malaysia.

### Susceptibility tests

Adult susceptibility tests were conducted using WHO standard kits provided by WHO (17). Twenty-five female mosquitoes aged 2–3 days fed on 10% sucrose solution during past night were exposed to DDT 4.0% and deltamethrin 0.05% impregnated papers for 60 minutes. The solvent-impregnated papers were used for the control group. There were 10 replicates for the treatment group and two replicates for the control one. After ending the exposure time, both mosquito groups were allowed to recover in holding tubes with a piece of cotton containing 10% sucrose solution on the top of tube for 24 hours and then the number of dead and live mosquitoes was counted. The susceptibility tests were separately repeated in the 3^rd^ and 6^th^ generations of *Cx. quinquefasciatus* in the insectary.

### Mosquito colonization

**Fig. 2.**
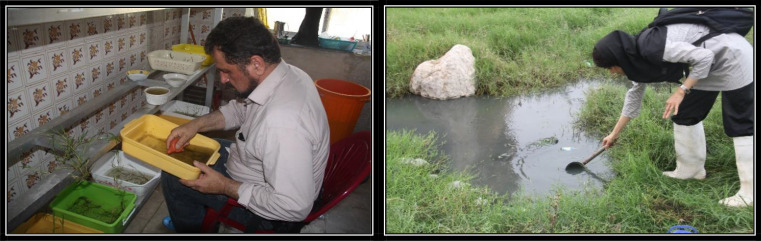
Collection of immature *Culex quinquefasciatus* from natural breeding places with floating Bermuda grass and subsequent establishment in the insectary of Bandar-Abbas Health Training and Research Station, Persian Gulf littoral, Iran

For the first time, a success was gained for establishment and colonization of *Cx. quinquefasciatus* in the water containing intact Bermuda grass “*Cynodon dactylon*” a perennial plant with spreading rhizomes and stolon. The plant was used directly in the immature breeding pans at natural state and without any chopping (Fig. 2). The adult mosquitoes were easily mated in a wooden netted cages dimensioned 30x 30x 30cm. The mosquitoes were blood-fed on a rooster, why the protein sources needed maturing the eggs.

### Statistical analysis

The mortality rate of *Cx. quinquefasciatus* after 24 hours recovery was calculated for each insecticide and generation and corrected using the Abbott’s formula if the mortality rate was 5–20% in the control group. The interpretation of the resistance level in *Cx. quinquefasciatus* was adopted from the WHO criteria and the terms resistant, suggestive of the existence of resistance and further investigation required, and susceptible were used for mortality rates <90%, 90–98% and >98%, respectively (17). Univariate analysis of variance (ANOVA) was applied after Arcsin transformation of the mortality rate to determine differences in mosquito mortality rates among generations. Significant differences of the mean of mortality rate were compared using Tukey or Games-Howell tests depending on the Post Hoc Test result. The significant difference in the mortality rate of *Cx. quinquefasciatus* between different generations was calculated using following formula: SE= √pq/n, where: “p” is the mortality rate, “q” is equal to 1 minus the mortality rate, and “n” is the sample size.

## Results

### Mass colonization

For the first time, a success was gained for establishment and colonization of *Cx. quinquefasciatus* in the water containing intact Bermuda grass “*Cynodon dactylon*” a perennial plant with spreading rhizomes and stolon. The plant was used directly in the immature breeding pans at natural state and without any chopping (Fig. 2). The adult mosquitoes were easily mated in a wooden netted cage dimensioned 30x 30cm. The mosquitoes were blood-fed on a rooster why the protein sources needed maturing the eggs.

### Bioassays

**Fig. 3.**
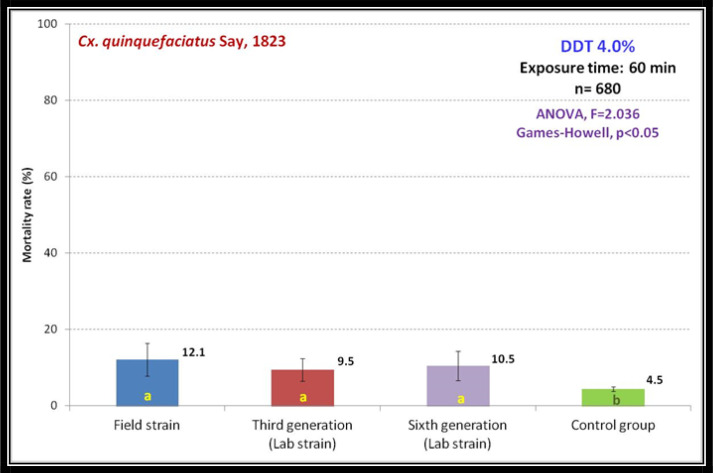
Comparison of susceptibility level of *Culex quinquefasciatus* exposed to DDT 4.0% among field population and subsequent generations gained from same population

Two-three days old female mosquitoes was exposed to DDT 4.0% and mortality rate of the field strain was 12.1%, compared to 9.5% and 10.5% in the 3rd (F3) and 6th (F6) generations. The mortalities between different generations exposed to DDT was not differed with each other, but with significant difference compared with control group (F= 2.036, p< 0.05) (Fig. 3).

**Fig. 4.**
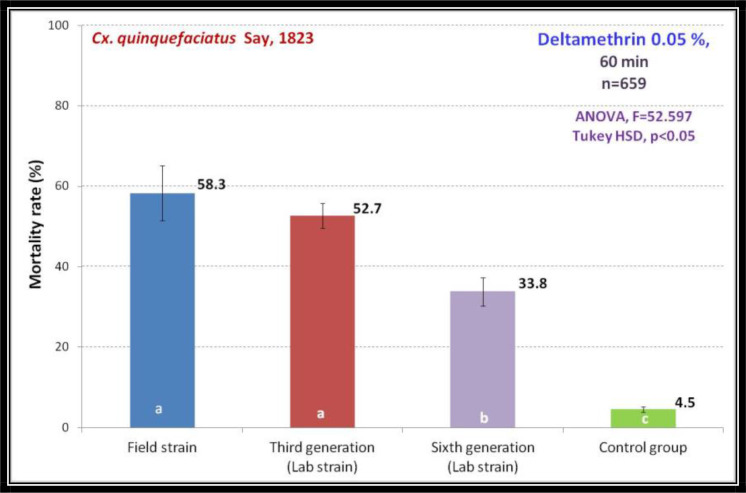
Comparison of susceptibility level of *Culex quinquefasciatus* to deltamethrin 0.05% on field population compared with lab strain achieved after mass colonization up to the six generations

The *Cx. quinquefasciatus* were revealed 58.3%, 52.7%, and 33.8% of mortality, when exposed 60min to deltamethrin 0.05% respectively for field strain, F3, and F6 generations. It was shown significant differences F3 compared to field strain and F6 mosquitoes and the mortality in control group showed significant differences with the treatment group (Fig. 4). The evidence showed that the subsequent mass colonization of a field strain, the susceptibility level will be unchangeable compared with the lab-colonized of the same population which undergone several mass-colonization which established the pyrethroid resistance.

## Discussion

Despite the confirmed role of *Cx. quinquefasciatus* as an arboviral vector in the subtropical climate, little attention has been paid to its bioecological characteristics in Iran. This is while huge densities of this species at larval and adult stages were observed at the urban area and extensive larval habitats could be found around the houses which its water originated from houses sewage. Moreover, pools and superficial wells at agricultural lands are potential larval habitats in the south and southeast Iran.

The susceptibility levels of female *Cx. quinquefasciatus* were tested according to a method recommended by the WHO. The results showed that this species was highly resistant to DDT and deltamethrin at diagnostic dose and 60min of exposure time. After the mass colonization of *Cx. quinquefasciatus*, the results of susceptibility tests were compared between field population and lab generations up to six generations achieved from the same field population to shown stability of resistance both for DDT and deltamethrin despite mass colonization up to six generations in the insectary condition. In this study, the susceptibility testing was followed from the latest WHO’s instruction (17), which indicated a 60min exposure time for all mosquito species and for specified diagnostic concentrations of insecticides, but according to the newly published WHO’s guidelines, the exposure time for DDT and *Culex* species were changed to 240min at the same discriminating concentration (18). Resistance-related studies of *Cx. quinquefasciatus* have been carried out for numerous insecticides around the world, from Asia to Europe, West Africa, and America. A high level of resistance to pyrethroids was found in Côte d’Ivoire and Burkina Faso, West Africa (8). There is a report of the resistance of *Cx. quinquefasciatus* to DDT and deltamethrin in Thailand caused by continuous use of insecticides for dengue vector control (19). In northeastern India, a study showed resistance to DDT and deltamethrin in *Cx. quinquefasciatus* collected from army cantonments and neighboring villages (20). There is a report of the resistance of *Cx. quinquefasciatus* to organochlorines, organophosphates, and pyrethroids in La Réunion Island, France (21). According to recent findings, resistance to DDT and pyrethroid in this species subsequently may lead to the inefficacy of the long-used insecticide-treated nets (10). This high resistance can be caused by several factors in the study area, one of which could be long-term use of DDT for combatting malaria vectors, especially in the south of Iran that goes back to half a century ago. Another factor may be the tendency of female gravid adults to lay eggs at sewage enriched with organic materials. Because of the blood-feeding tendency of *Cx. quinquefasciatus* towards the poultries and wild birds, which are considered as potential reservoirs of arboviral agents, the risk of the transmission of these diseases to humans increases in the subsequent blood-feeding sessions. This vector is exposed to the chemicals that may be present in the urban wastewaters. Cross-resistance should also be considered as another factor for inactivation of pyrethroid insecticides. The target of organochlorines (DDT) and synthetic pyrethroids is the sodium channels of the nerve sheath (22). Therefore, due to the similar target site, cross-resistance may develop and resistance to one of these insecticides may make mosquitoes resistant to the other one. The findings of present study may reflect the role of the kdr gene in resistance to organochlorine which has cross-resistance with pyrethroid insecticides. Furthermore, another finding is irreversible and progressive trends of resistance intensity to the pyrethroids among the colonized mosquitoes reared for several generations. This is the first study of the resistance status of *Cx. quinquefasciatus* in Iran.

## Conclusion

The findings indicated the stability of DDT resistance in the field population of *Cx. quiquefasciatus* as well as among lab-bread of same population. The deltamethrin resistance showed progressive trend in the lab compared with wild population after multiple colonization in the insectary. Hence, precise control management should be spotted for efficacy of control operations against *Culex* vectors.
